# Vascular Effects of Urocortins 2 and 3 in Healthy Volunteers

**DOI:** 10.1161/JAHA.112.004267

**Published:** 2013-02-22

**Authors:** Sowmya Venkatasubramanian, Megan E. Griffiths, Steven G. McLean, Mark R. Miller, Rosa Luo, Ninian N. Lang, David E. Newby

**Affiliations:** 1 Centre for Cardiovascular Science, University of Edinburgh, Edinburgh, United Kingdom (S.V., M.E.G., S.G.M.L., M.R.M., N.N.L., D.E.N.); 2 Neurocrine Biosciences Inc, San Diego, CA (R.L.)

**Keywords:** forearm plethysmography, nitric oxide, urocortin 2, urocortin 3, vasodilatation

## Abstract

**Background:**

Urocortin 2 and urocortin 3 are endogenous peptides with an emerging role in cardiovascular pathophysiology. We assessed their pharmacodynamic profile and examined the role of the endothelium in mediating their vasomotor effects in vivo in man.

**Methods and Results:**

Eighteen healthy male volunteers (23±4 years) were recruited into a series of double‐blind, randomized crossover studies using bilateral forearm venous occlusion plethysmography during intra‐arterial urocortin 2 (3.6 to 120 pmol/min), urocortin 3 (1.2 to 36 nmol/min), and substance P (2 to 8 pmol/min) in the presence or absence of inhibitors of cyclooxygenase (aspirin), cytochrome P450 metabolites of arachidonic acid (fluconazole), and nitric oxide synthase (L‐NMMA). Urocortins 2 and 3 evoked arterial vasodilatation (*P*<0.0001) without tachyphylaxis but with a slow onset and offset of action. Inhibition of nitric oxide synthase with L‐NMMA reduced vasodilatation to substance P and urocortin 2 (*P*≤0.001 for both) but had little effect on urocortin 3 (*P*>0.05). Neither aspirin nor fluconazole affected vasodilatation induced by any of the infusions (*P*>0.05 for all). In the presence of all 3 inhibitors, urocortin 2– and urocortin 3–induced vasodilatation was attenuated (*P*<0.001 for all) to a greater extent than with L‐NMMA alone (*P*≤0.005).

**Conclusions:**

Urocortins 2 and 3 cause potent and prolonged arterial vasodilatation without tachyphylaxis. These vasomotor responses are at least partly mediated by endothelial nitric oxide and cytochrome P450 metabolites of arachidonic acid. The role of urocortins 2 and 3 remains to be explored in the setting of human heart failure, but they have the potential to have major therapeutic benefits.

**Clinical Trial Registration:**

http://www.clinicaltrials.gov//. Unique identifier: NCT01096706 and NCT01296607.

## Introduction

Urocortin peptides, especially urocortins 2 and 3, have prominent cardiovascular roles and are expressed in the heart. Although related to corticotrophin‐releasing hormone (CRH), they do not appear to have any role in the regulation of the hypothalamic‐pituitary‐adrenal axis.^1–2^ The effects of CRH and the urocortins are mediated via 2 G‐protein‐coupled receptors: CRH‐R1 and CRH‐R2. Although CRH‐R1 is predominantly expressed in the brain and not in the heart, CRH‐R2 is expressed in the myocardium and vascular smooth muscle.^2–3^ It is found in human coronary artery microvascular endothelial cells and has been detected in the endothelium of a variety of peripheral vascular beds. Urocortin 1 activates both receptors, whereas urocortins 2 and 3 are potent selective agonists at CRH‐R2 but have no effect on CRH‐R1.^4^

The role of urocortins in cardiovascular physiology and pathophysiology, particularly heart failure, has become increasingly apparent. Intravenous urocortin 1 causes marked vasodilatation in mice via CRH‐R2.^5–6^ Furthermore, mice lacking CRH‐R2 receptors are hypertensive, suggesting a role for urocortin in the maintenance of basal vascular tone.^6^ Systemic administration of urocortin 2 in humans increases cardiac output, heart rate, and left ventricular function while decreasing systemic vascular resistance, and these effects may be amplified in the setting of heart failure.^7–8^ Urocortin 3 has not previously been administered to humans, but in an ovine model, both urocortin 2 and urocortin 3 appeared to produce similar cardiovascular effects.^9–10^ Although urocortin 2 and urocortin 3 each activate the same receptor, potential differences in their cardiovascular therapeutic utility may arise from their differing pharmacokinetic and pharmacodynamic profiles.^1^

The cardiovascular responses of urocortins represent an amalgamation of systemic actions. However, their direct in vivo arterial vasomotor effects have never been examined in humans. Moreover, the role of the endothelium in the mediation of these responses is unknown. Therefore, our study's aims were to conduct the first comparative clinical assessment of local arterial vasomotor effects of urocortins 2 and 3 and to determine the role of the endothelium in the mediation of these effects.

## Methods

All studies were approved by the local research ethics committee and carried out in accordance with the Declaration of Helsinki. Written informed consent was obtained from all participants prior to the study.

### Study Participants

Eighteen healthy nonsmoking male volunteers were recruited into the series of vascular studies. Participants had no documented medical history, were taking no regular medications, and tested negative in a urinary toxicology screen (Nova Test, One Step Diagnostic Rapid Test, CA) for recreational drugs.

### Vascular Studies

All studies were conducted using a double‐blind, randomized, controlled crossover design. They were performed with the patient lying supine in a quiet, temperature‐controlled room (22°C to 25°C). Volunteers fasted for 4 hours prior to the study and refrained from alcohol and caffeine for 24 hours prior to the study. Venous cannulae (17G) were inserted into large subcutaneous veins in the antecubital fossae of both arms at the start of the study to facilitate periodic venous sampling. In view of the theoretical risk of alterations in body temperature^11^ and blood glucose concentrations^12^ with the first‐in‐human administration of urocortin 3, tympanic temperature (Genius 2 Tympanic Thermometer, Coviden, Boston, MA) and capillary blood glucose (Advantage Accucheck blood glucometer, USA) measurements were performed at baseline and after each dose of urocortin 3. Heart rate and blood pressure were monitored at regular intervals throughout the study with a semiautomated oscillometric sphygmomanometer (Omron 705IT).

Subjects underwent brachial artery cannulation in the nondominant forearm with a 27 standard wire‐gauge steel needle. Forearm blood flow was measured in the infused and noninfused forearms using bilateral venous occlusion plethysmography as described previously.^13–14^

### Pharmacodynamic Study

**Figure 1.**
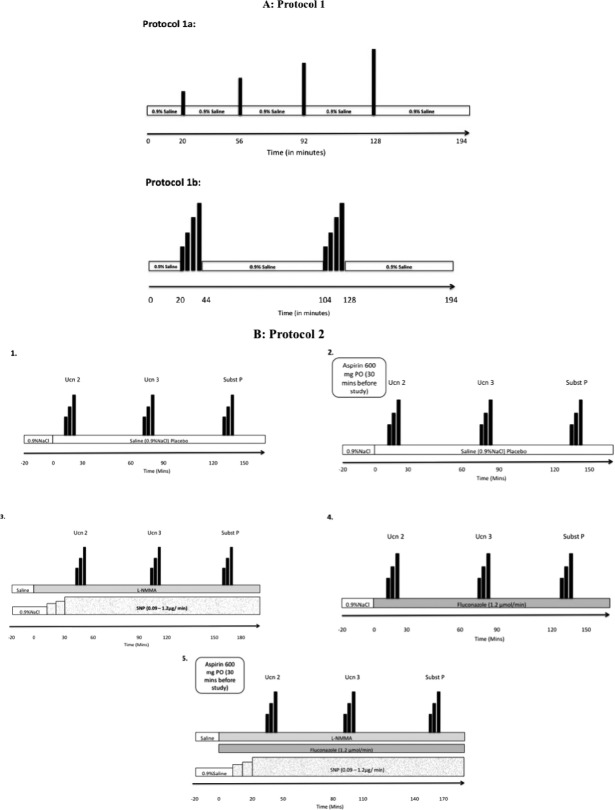
Schematic representation of study protocols. A, Protocol 1—incremental intra‐arterial doses of urocortin 2 (Ucn 2; 3.6 to 120 pmol/min) and urocortin 3 (Ucn 3; 1.2 to 36 nmol/min) in the presence (protocol 1a) and absence (protocol 1b) of saline washout. B, Protocol 2—incremental intra‐arterial infusions of Ucn 2 (3.6 to 36 pmol/min), Ucn 3 (1.2 to 12 nmol/min), and substance P (sub P; 2 to 8 pmol/min) in the presence of (1) saline placebo, (2) oral aspirin, (3) “nitric oxide” clamp, (4) intra‐arterial fluconazole, and (5) a combination of oral aspirin, fluconazole, and nitric oxide clamp. L‐NMMA indicates L‐N(G)‐monomethyl arginine citrate.

Eight healthy volunteers attended on 4 occasions (protocol 1) separated by at least 1 week (Figure 1A). After an initial infusion of normal saline (0.9%) for 20 minutes, volunteers received discontinuous (protocol 1a) or continuous (protocol 1b) incremental intra‐arterial doses of urocortin 2 (3.6 to 120 pmol/min; Neurocrine Biosciences, Inc, San Diego, CA) or urocortin 3 (1.2 to 36 nmol/min; GenScript, NJ) interspersed with saline infusions between doses as appropriate.

### Mechanistic Study

An additional 10 healthy volunteers (protocol 2) attended on each of 5 occasions to receive incremental intra‐arterial doses of urocortin 2 (3.6 to 36 pmol/min), urocortin 3 (1.2 to 12 nmol/min), and substance P (2 to 8 pmol/min; a control endothelium‐dependent vasodilator that evokes endothelial tissue plasminogen activator release; Clinalfa Basic, Bachem Distribution Services Gmbh, Germany) (Figure 1B). These infusions were administered in the presence of (1) a placebo, (2) oral aspirin 600 mg (cyclo‐oxygenase inhibition), (3) a “nitric oxide clamp” (nitric oxide synthase inhibition; see below), (4) intra‐arterial fluconazole (1.2 μmol/min; inhibition of cytochrome P450 metabolites of arachidonic acid), and (5) a combination of oral aspirin, intra‐arterial fluconazole, and the nitric oxide (NO) clamp.

The nitric oxide clamp was used to determine the contribution of nitric oxide to urocortin‐induced vasodilatation. Following baseline saline infusion, the nitric oxide synthase inhibitor L‐N(G)‐monomethyl arginine citrate (L‐NMMA; 8 μmol/min; Clinalfa Basic, Bachem Distribution Services Gmbh, Germany) was infused intra‐arterially. To compensate for L‐NMMA‐induced basal vasoconstriction, forearm blood flow was returned to baseline using a titrated dose of the exogenous nitric oxide donor sodium nitroprusside (SNP; 90 to 1200 ng/min; Hospira Inc, Lake Forest, IL). Once baseline blood flow had been restored, this dose of SNP was coinfused with L‐NMMA and continued throughout the study. This arrangement allows a constant “clamped” delivery of exogenous nitric oxide while endogenous nitric oxide synthase activity is abolished.

The order of urocortin 2, urocortin 3, and substance P infusions was randomized between subjects but kept constant for all visits of each individual subject. The order of infusion of inhibitors was also randomized in a double‐blind manner.

### Venous Sampling

Blood sampling was carried out at baseline for the assessment of full blood count, liver and renal function tests, cholesterol, and blood glucose levels. Analysis was performed by the local clinical biochemistry and hematology reference laboratories.

### Data Analysis and Statistics

Forearm blood flow data were analyzed as described previously.^13^ A normal distribution of the data was demonstrated using the D'Agostino & Pearson omnibus normality test. All variables are reported as mean±SEM using repeated‐measures analysis of variance (ANOVA) with post hoc Bonferroni corrections and a 2‐tailed Student *t* test as appropriate (Graph‐Pad Prism, GraphPad Software, San Diego, CA). Significance was taken as 2‐sided *P*<0.05.

## Results

### Study Participants

All volunteers were young healthy men (23±4 years). Both urocortin 2 and urocortin 3 produced marked localized flushing in the infused arm along with facial flushing at the highest doses. Volunteers also experienced heightened awareness of their heartbeat during and immediately after the highest dose of urocortin 3 (36 nmol/min). All symptoms were self‐limiting, well tolerated, and short‐lived. Substance P also induced localized flushing of the infused forearm, which was self‐limiting. There were no clinically significant changes in the standard hematological and biochemical analytes including full blood count, blood glucose, cholesterol, and renal and hepatic function throughout the study (data on file). Capillary blood glucose and body temperature (tympanic) remained unchanged during all doses of urocortin 3 (data on file).

**Figure 2.**
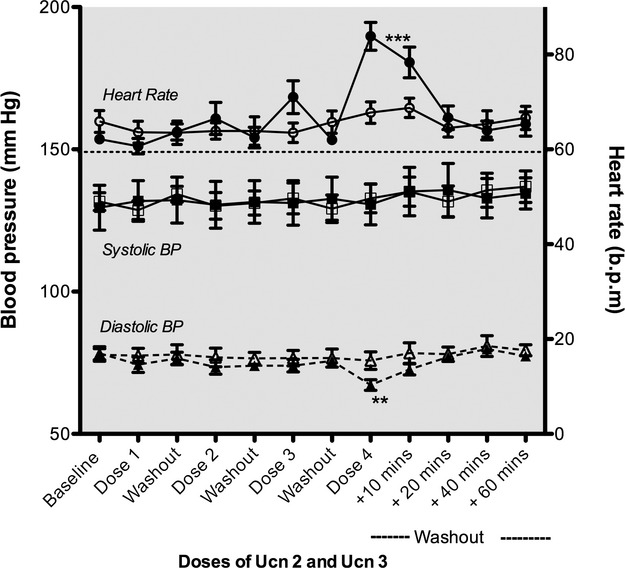
Hemodynamic responses to intra‐arterial infusion of urocortin 2 (Ucn 2; 3.6 to 120 pmol/min) and urocortin 3 (Ucn 3; 1.2 to 36 nmol/min). At a dose of 36 nmol/min, Ucn 3 evoked transient tachycardia associated with a drop in diastolic blood pressure. Open symbols, Ucn 2; closed symbols, Ucn 3; circle, heart rate; square, systolic blood pressure (BP); triangle, diastolic BP; ***P<0.0001; **P=0.004; dose 1=3.6 pmol/min Ucn 2 or 1.2 nmol/min Ucn 3; dose 2=12 pmol/min Ucn 2 or 3.6 nmol/min Ucn 3; dose 3=36 pmol/min Ucn 2 or 12 nmol/min Ucn 3; dose 4=120 pmol/min Ucn 2 or 36 nmol/min Ucn 3; bpm indicates beats per minute.

Systolic blood pressure and noninfused forearm blood flow remained unchanged at all doses with all 3 peptides across both protocols. However, at the highest infused dose of urocortin 3 (36 nmol/min), there was a sinus tachycardia (+22±2 beats/min; ANOVA, *P*<0.0001) with an associated drop in diastolic blood pressure (−8.5±0.8 mm Hg; ANOVA *P*=0.004; Figure 2) that was not seen with either substance P or urocortin 2 infusions.

### Pharmacodynamic Effects of Urocortins 2 and 3

**Figure 3.**
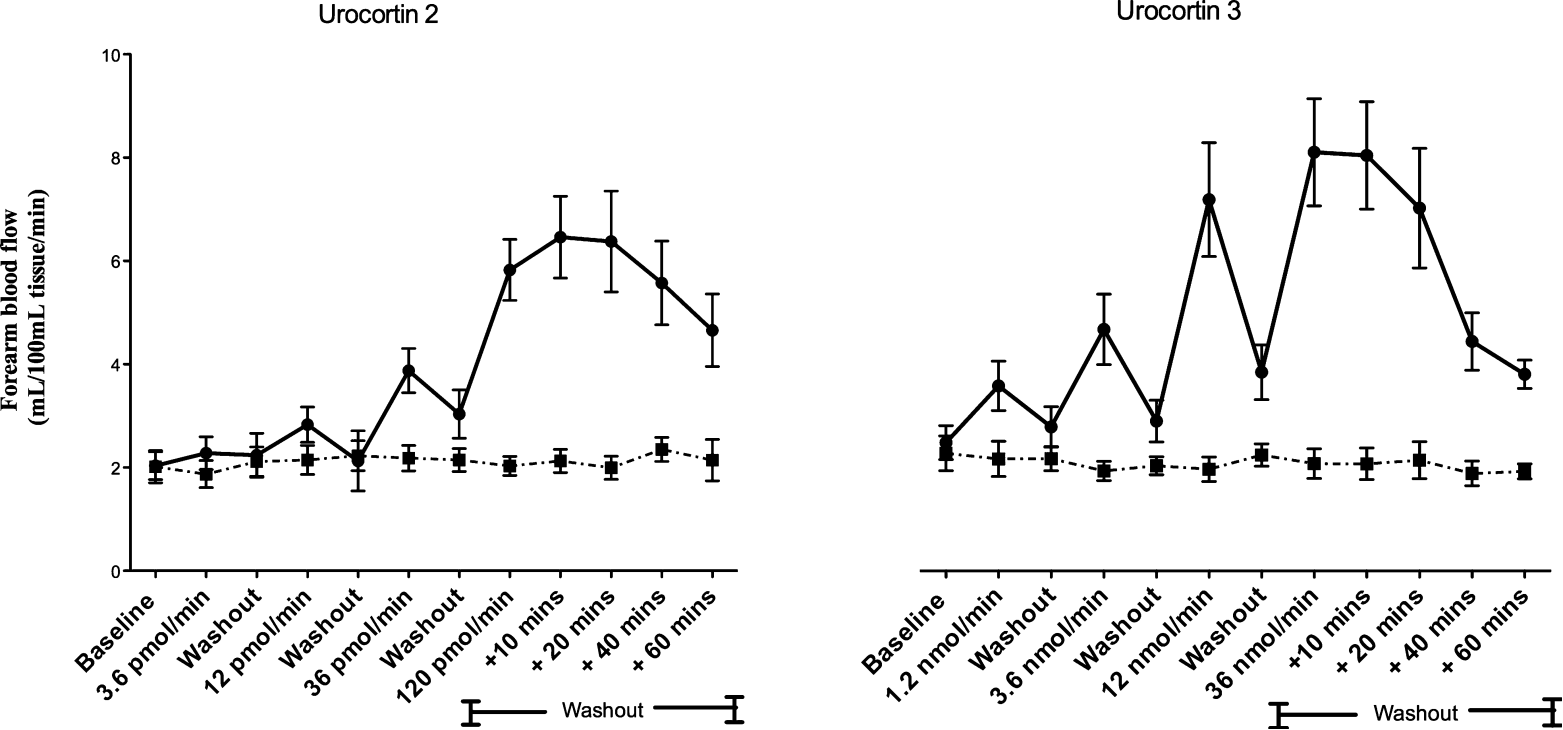
Forearm arterial blood flow responses to increasing doses of urocortin 2 (Ucn 2) and urocortin 3 (Ucn 3). Circle, infused forearm blood flow; square, noninfused forearm blood flow. *P*<0.0001 at all doses.

Both urocortin 2 and urocortin 3 evoked dose‐dependent arterial vasodilatation in the infused arm (2‐way ANOVA, *P*<0.0001) (Figure 3).

**Figure 4.**
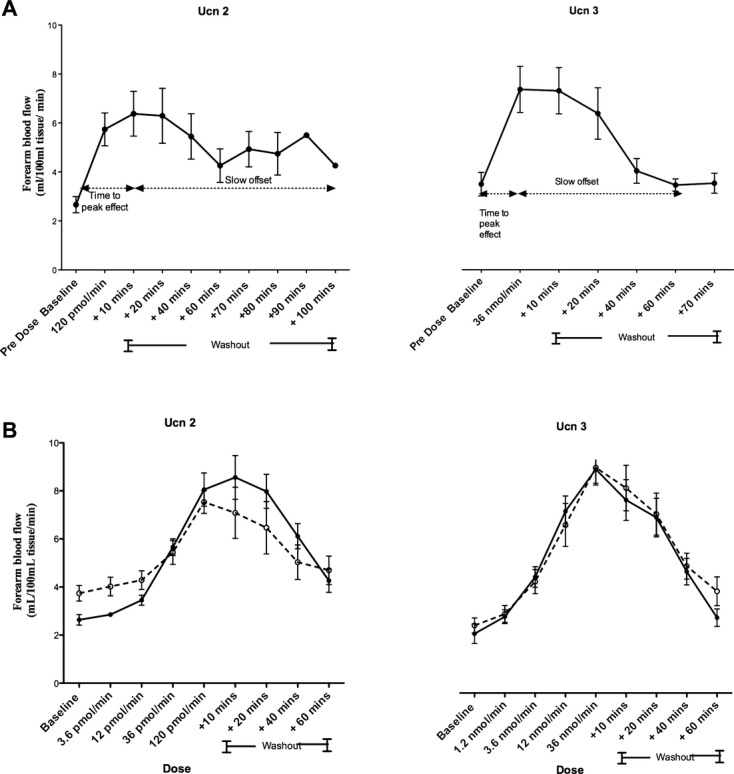
Pharmacodynamics of urocortin 2 (Ucn 2) and urocortin 3 (Ucn 3). A, Onset and offset of vasodilatory effect of Ucn 2 (left) and Ucn 3 (right) after infusion of highest dose. B, Within‐day reproducibility of Ucn 2 (left) and Ucn 3 (right); *P*=nonsignificant, first dose response vs second dose response; Ucn 2 and Ucn 3. Closed circle: first dose response; open circle, second dose response.

Maximal vasodilatation with urocortin 2 was apparent ≈10 minutes after cessation of the 120 pmol/min infusion (paired Student *t* test, Ucn 2 120 pmol/min versus +10‐minute washout; *P*=0.04), and thereafter the blood flow gradually returned toward baseline. This was in contrast to the effect seen with urocortin 3, for which the maximum vasodilatory response was immediate (Figure 4A). The offset of vasodilatation was prolonged with both peptides, although urocortin 2 took longer than urocortin 3 to return to baseline (Figure 4A).

The vasodilator effects of both peptides appeared to be reproducible within a day, with no evidence of tachyphylaxis (2‐way ANOVA, *P*>0.05 for all; Figure 4B).

### Endogenous Fibrinolytic Factors

Preliminary data showed no effect of urocortin 2 or urocortin 3 on endothelial release of tissue plasminogen activator and plasminogen activator inhibitor‐1 (data on file).

### Mechanism of Vasodilatation

**Figure 5.**
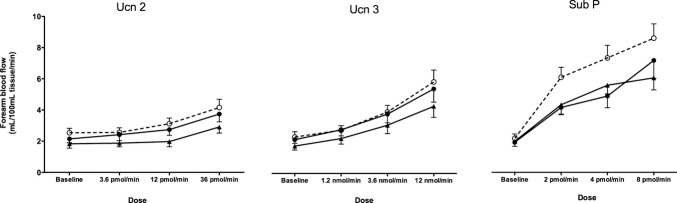
Vasomotor effects of inhibition of endothelial nitric oxide synthase, cycloxygenase, and cytochrome P450 metabolites of arachidonic acid on urocortin 2–, urocortin 3–, and substance P–mediated vasodilatation. Open circle, placebo; closed circle, nitric oxide clamp; closed triangle, combined aspirin (600 mg), nitric oxide clamp, and fluconazole (1.2 μmol/min).

Baseline forearm arterial blood flow was unaffected by oral aspirin or intra‐arterial fluconazole, and the coinfusion of SNP restored baseline blood flow during L‐NMMA administration (2‐way ANOVA, *P*>0.05 for all). Inhibition of nitric oxide synthase reduced arterial vasodilatation to substance P and urocortin 2 (2‐way ANOVA, *P*≤0.001 for both) but had no apparent effect on urocortin 3–induced vasodilatation (2‐way ANOVA, *P*=0.36). Neither inhibition of cyclo‐oxygenase with aspirin nor cytochrome P450 metabolites of arachidonic acid with fluconazole affected the vasodilatation induced by the urocortins or substance P (2‐way ANOVA, *P*>0.05 for all; data on file). In the presence of all 3 inhibitors, substance P–, urocortin 2–, and urocortin 3–induced vasodilatation was further attenuated (2‐way ANOVA, *P*<0.001 for all) but not completely abolished. Combined inhibition of cyclo‐oxygenase, nitric oxide synthase, and cytochrome P450 metabolites of arachidonic acid produced a greater reduction in vasodilatation than the nitric oxide clamp alone (2‐way ANOVA, *P*≤0.005 for urocortins 2 and 3; Figure 5).

## Discussion

This study represents the first administration in humans of urocortin 3 and demonstrates that both urocortin 2 and urocortin 3 directly evoke potent and prolonged arterial vasodilatation that is, at least in part, mediated by the endothelium. These findings are of direct relevance not only to our understanding of human cardiovascular physiology but also inform the development of therapies targeting the urocortin system for the treatment of conditions such as heart failure.

The forearm arterial vasodilator effects of urocortin 2 and urocortin 3 are consistent with data from in vitro^3,15–18^ and preclinical^19–20^ animal studies. However, in contrast with existing preclinical data, we observed a more marked difference in potency between the 2 peptides. Although preclinical studies have suggested urocortin 2 is 10‐fold more potent,^21–22^ Wiley et al^3^ showed equipotency of urocortins 2 and 3 in isolated human internal mammary arterial segments. In contrast, here we observed that a 300‐fold‐higher dose of urocortin 3 was required to evoke comparable vasomotor effects in human forearm arterial circulation. This discrepancy underlines the importance of a direct head‐to‐head assessment in vivo in humans, without which the extrapolation of preclinical data may be deceptive.

Urocortins 2 and 3 are specific agonists at the G‐protein‐coupled CRH‐R2 receptors, mediating their effects through a cascade of intracellular signaling pathways including adenyl cyclase, cyclic adenosine monophosphate,^23–24^ and mitogen‐activated protein kinases.^25^ Other well‐characterized G‐protein‐coupled receptor agonists such as bradykinin,^26–27^ substance P,^28^ and protease activated receptor type 1 activating peptide^29–30^ evoke vasodilatation with rapid onset and offset in human forearm arterial circulation. Unlike these agonists, the maximal vasodilator effect evoked by urocortin 2 in this study was apparent ≈10 minutes following completion of the highest dose (protocol 1a). In addition to a late maximal response with urocortin 2, we also observed a prolonged offset. Even 100 minutes post–urocortin 2 administration, infused forearm blood flow remained elevated. Although not as lengthy as the effect evoked by urocortin 2, urocortin 3–evoked vasodilatation was also prolonged and took 1 hour for blood flow to return to baseline after discontinuation of the infusion. This prolonged offset of effect is unusual for G‐protein‐coupled receptor agonists, although a similar time course has been observed in response to apelin^31^ and vasopressin,^32^ and is thought to be the result of prolonged receptor occupancy. In vitro studies by Hoare et al^33^ have demonstrated differing affinities of urocortin 2 and urocortin 3 to the CRF‐R2 receptor determined by the affinity of the extracellular domains of the CRF receptors to these agonists. It remains to be established whether urocortin 2 induces receptor transformation, thereby promoting prolonged binding to CRH‐R2 and a delayed maximal response. However, when assessed in isolation, it is clear that the direct vasomotor effects of urocortin 2 and urocortin 3 are more prolonged than previously reported.

The vasodilator effects of both peptides showed good within‐day reproducibility without evidence of tachyphylaxis. These are important properties, especially for potential applications in extended or chronic therapies in which predictable and reproducible pharmacologic and hemodynamic effects are needed.

Several mechanisms have been proposed to explain the mechanistic pathways of urocortin‐mediated vasorelaxation. Studies to date have suggested that the mechanism involved may depend on the species or vascular bed in question. In rats, both endothelium‐dependent^17,34^ and ‐independent components were implicated.^35^ Grossini et al^36^ demonstrated that urocortin 2–mediated vasorelaxation in the coronary arteries of anaesthetized pigs was mediated by nitric oxide. However, urocortin‐mediated vasodilatation appeared to be independent of endothelial integrity in isolated human coronary and internal mammary artery segments.^3,16^ In the current study, the NO clamp appeared to cause modest inhibition of urocortin 2–mediated vasodilatation and appeared to be marginally more pronounced with urocortin 2 compared with urocortin 3. Inhibition of cytochrome P450 metabolites of arachidonic acid with fluconazole alone did not have an appreciable effect on urocortin‐mediated vasodilatation, but its addition enhanced the inhibitory effect of the NO clamp. This suggests that endogenous nitric oxide and cytochrome P450 metabolites of arachidonic acid may have a close interrelationship and can compensate for one another to maintain vascular tone. A similar effect has previously been described for endothelium‐derived hyperpolarizing factor (EDHF), whereby its relative importance increases in the face of impaired NO bioavailability under conditions of oxidative stress. Although urocortin 2– and urocortin 3–mediated vasodilatation was inhibited by the combination of all 3 inhibitors, it was not abolished, and a substantial degree of vasomotor activity remained. We cannot exclude a contribution from other endothelial pathways such as residual “fluconazole‐insensitive” EDHF, although the results do suggest a substantial contribution from direct smooth muscle activation. Although the role of the endothelium is substantial, a large contribution from endothelium‐independent vasodilator mechanisms may be seen as an advantage in the potential therapeutic applications of CRH‐R2 agonism. Arguably, this would allow a more predictable response from the manipulation of urocortin (2 or 3) as a therapy for cardiovascular conditions, the vast majority of which would be expected to be associated with impaired endothelial homeostatic mechanisms but preserved smooth muscle activity.

This study was designed to assess locally active, subsystemic doses of urocortins 2 and 3 in forearm arterial vasculature. Indeed, the forearm venous occlusion plethysmography model allows these local effects to be studied at doses 10‐ to 100‐fold lower than that usually expected or required to elicit a systemic response. It would appear, however, that there was a degree of systemic overspill associated with the top dose of urocortin 3 tested. The observed decrease in diastolic blood pressure and increase in heart rate observed during and after the infusion of the top dose of urocortin 3 is notable, particularly as it occurred in the absence of any change in blood flow in the contralateral noninfused forearm. Usually, in the case of systemic overspill, a corresponding change in the “control” noninfused arm would be observed,^28^ but this was not the case with the infusion of the dose of urocortin 3. We therefore hypothesize that the hypotensive effect observed reflects vasodilatation in another, more sensitive vascular bed, such as splanchnic circulation, with consequent reflex tachycardia. Absence of a similar hemodynamic response to the highest dose of urocortin 2 may be the result of lower subsystemic doses of the less potent peptide urocortin subtype or, conceivably, a differential in the relative sensitivity of splanchnic and forearm arterial circulation to urocortin 2. However, systemic doses up to 100 μg of urocortin 2 have been shown to increase heart rate and decrease diastolic blood pressures in healthy adults.^7^

### Study Limitations

The size of our study population was relatively small. However, we have previously described the influence of a range of factors on blood flow in forearm vasculature using sample sizes of ≤12 subjects^28,30–31,37,29,38–39^ As with most other physiological studies, we have not performed multiple testing correction for the data obtained.

We studied the changes in forearm blood flow during brief local intrabrachial infusions of urocortins 2 and 3. The apparent differences between urocortin 2 and urocortin 3 observed in our study may reflect, in part, differences between the 2 peptides in the tissue exposure achieved.

We do not as yet know the effects of prolonged infusions of these peptides. Moreover, the effects of urocortins 2 and 3 in patients with heart failure remain to be explored. Although there is good concordance between the vasomotor responses observed in the forearm resistance vessels and other vascular beds,^40^ further studies will be required to explore their systemic effects in health and disease in humans.

## Conclusions

We have demonstrated that urocortin 2 and urocortin 3 evoke potent prolonged arterial vasodilatation and that their effects are at least partly dependent on endothelial nitric oxide and cytochrome P450 metabolites of arachidonic acid. These data provide important insights into human cardiovascular physiology, and they will inform the development of further therapies directed toward the urocortin pathway. The in vivo role of this endogenous peptide system in patients with heart failure and the role of CRH‐R2 in human health and disease remain to be explored.
